# Atmospheric Corrosion of Different Steel Types in Urban and Marine Exposure

**DOI:** 10.3390/ma17246211

**Published:** 2024-12-19

**Authors:** Luca Paterlini, Andrea Brenna, Federica Ceriani, Matteo Gamba, Marco Ormellese, Fabio Bolzoni

**Affiliations:** Department of Chemistry, Materials and Chemical Engineering “Giulio Natta”, Politecnico di Milano, Via Mancinelli 7, 20131 Milan, Italy; luca.paterlini@polimi.it (L.P.); andrea.brenna@polimi.it (A.B.); federica.ceriani@polimi.it (F.C.); matteo.gamba@polimi.it (M.G.); marco.ormellese@polimi.it (M.O.)

**Keywords:** atmosphere, corrosion, carbon steel, stainless steel, galvanized steel, marine, urban

## Abstract

The aim of the present work is to study the atmospheric corrosion behavior of metals exposed to both urban (Milan, IT-Lombardia) and marine (Bonassola, IT-Liguria) atmospheres in Italy. A number of coupons (100 × 150 mm) of carbon steel (CS), hot-dip galvanized steel (GS) and different grades of stainless steel (SS) were exposed. At fixed periods of time, samples were characterized by means of Linear Polarization Resistance (LPR), mass loss tests and corrosion product analysis. The corrosion rate on carbon steel exposed to an urban atmosphere, obtained by means of mass loss tests and LPR, are in good agreement with the value estimated by the dose–response function according to the ISO 9223 standard. The yielded results can be classified in corrosivity class C2 of the same ISO 9223. Similar measurements on galvanized steel exhibited a coherent average corrosion rate. Higher corrosion rates were measured for samples exposed to a marine atmosphere for both materials, with values belonging to exposure classes C4-C5 for both materials. Stainless steel samples exhibited only superficial staining in the case of marine exposure, even after just a few months.

## 1. Introduction

Metals are subject to corrosion when exposed to atmospheric environmental conditions, and to correctly design outdoor structures to outlive their design life, an extensive knowledge of their corrosion behavior over the years is then required.

For these reasons, the atmospheric corrosion of metals is a prosperous research field [1,2]: several international research initiatives [3,4] produced a large amount of data during the last decades of the 20th century, leading to the formation of an ISO standard set [5,6,7,8], last revised in 2012.

The standards address the atmospheric corrosion attack of the most used metals (carbon steel, zinc, copper and aluminum) through two main approaches:

- Based on first-year measurement of standardized specimens’ corrosion rate [9];
- Based on environmental information through the dose–response function that takes into account relative humidity, temperature, pollution by sulfur dioxide and airborne salinity.

The standard defines a set of aggressiveness classes related to the most relevant factors influencing atmospheric corrosion, such as time of wetness, sulfur oxides and chlorides concentration. According to the first-year corrosion rate, the ISO standard [5] classifies the corrosion severity into six aggressiveness classes, ranging from C1 to CX (Table 1).

Once the first-year corrosion rate is measured or calculated through the dose–response function, the corrosion attack (*D*) for the first 20 years can be estimated according to the following equation:

(1) $$D=r_{corr}t^{b}$$

where *t* is the exposure time; *r_corr_* is the first-year corrosion rate expressed in thickness loss [μm/a] or weight loss [g/(m^2^a)]; and *b* is the metal–environment-specific time exponent, that usually ranges between 0.5 and 1.

According to the ISO standard, after the 20th year of exposure, the corrosion rate is to be considered constant and equivalent to the 20th year corrosion rate for all the following years, as the corrosion product layer thickness is stabilized, along with its protective effects [6].

Considerable uncertainty is associated with determining corrosivity categories, as well as the corrosion rate estimation once the exposure class is selected [5].

Data consistency is therefore essential to reliably predict corrosion behaviors. Across Europe, open research data collection of metal corrosion rates is constantly taking place at the exposure sites officially recognized in the “Exposure Site Catalogue” of the European Federation of Corrosion, EFC [10].

The aim of the present study is to provide exhaustive corrosion rate data for selected carbon steel (CS), galvanized steel (GS) and stainless steel (SS) exposed to urban and marine environments for up to 26 months.

Carbon steels are the most widely used materials for structural purposes, with low alloying element content and costs. They are engineered to optimize the mechanical properties-to-cost ratio. Carbon steels are susceptible to general corrosion, with a corrosion rate up to few hundreds µm/y, depending on environmental parameters such as time of wetness [2,11,12,13], chloride deposition rate, temperature, etc. The rust layer that forms on bare carbon steel during the wet corrosion process is a complex equilibrium of Fe-oxides and Fe-hydroxides whose composition may vary with pollutant and chloride concentration [14,15,16,17,18,19]. The rust layer is only mildly protective, as confirmed by Misawa et al. [14], but not adherent, leading to its periodic detachment from the metal surfaces, offering no lasting protection [20]. Therefore, carbon steel should not be used in bare conditions, if not only in extremely arid climates [11].

Galvanized steels are steels coated with zinc through hot-dip or electrodeposition processes to provide enhanced corrosion resistance [21]. Zinc is an amphoteric metal, which forms a passive layer of Zn-carbonates in the atmosphere, providing good corrosion resistance. When galvanically coupled with steel, it behaves as an anode, preserving the underlying steel surface even when the coating is locally damaged. The zinc coating of galvanized steel produced through the hot-dip process reaches a thickness of up to 100 µm [2,22], with corrosion rates ranging between 0.1–0.7 µm/y in C1 exposure and 4.2–8.4 µm/y in C5 environments. Therefore, the zinc corrosion rate is usually 10–30 times lower than that of carbon steel in an equal environment [2,5]. Long-term exposure tests (over 10 years) of galvanized steel specimens, following EN ISO 8565:2011 [9], have confirmed the corrosion rates proposed by standards across several European locations, highlighting an approximately linear correlation between zinc thickness loss and exposure time, particularly in industrial and urban environments [21,22,23].

Stainless steels, according to EN 10088-1:2023, are ferrous alloys with a chromium content greater than 10.5% by weight [24], which is essential for the formation of a nanometric, highly protective chromium oxide layer, responsible for their enhanced corrosion resistance [25,26]. Unlike CS and GS, SS exhibits a broader range of corrosion behaviors when exposed to the atmosphere and is sensitive to pollutants and chlorides, experiencing localized corrosion attacks whenever the passive layer breaks down [25,27,28,29]. The most relevant localized corrosion attacks affecting SS are pitting and crevice corrosion, which have been extensively studied in the last decades, knowledge carefully summarized and reviewed by Sedriks and Szklarska [29,30].

In an atmospheric environment, SS corrosion is mostly an aesthetic issue, often referred to as “staining”, rather than a threat to the integrity of the material itself. Pit growth is indeed often limited to tenths of µm/a [31], except for particularly unfavorable geometries that lead to stagnant conditions, which is of course not the case in standard exposure conditions [24].

Chemical composition is the primary factor influencing the pitting resistance of a SS, directly contributing to the stability of the passive oxide film in aggressive environments [30,32,33,34]. The Pitting Resistance Equivalent Number, better known as PREN, summarizes in a single equation (Equation (2)) the beneficial effects of relevant alloying elements, assigning specific weight to each of them depending on their contribution to pitting initiation resistance.

(2) $$PREN=\left[Cr\%\right]+3.3\;\left[Mo\%\right]+16\;[N\%]$$

A higher *PREN* is empirically associated with higher pitting corrosion resistance in these alloys.

However, other parameters may affect the breakdown resistance of the passive layer, including environmental ones, such as pH [28,30], and process ones, such as cold working [27,35] and surface finishing [35,36,37,38].

To achieve data consistency regarding metal corrosion rates in atmosphere for CS and GS, which suffer generalized corrosion, measurements are typically conducted through gravimetric analysis in accordance with EN ISO 8565:2011 [9] and further validated through electrochemical techniques, i.e., Linear Polarization Resistance (LPR) [39,40,41], Electrochemical Impedance Spectroscopy (EIS) [40,42] and Electrochemical Frequency Modulation (EFM) [43,44,45]. SSs, which suffer localized corrosion phenomena and are not included in the ISO standard abovementioned, are evaluated for their corrosion resistance usually by visual inspection and pit depth measurement [46]. Scarce weight loss over the years and the highly resistive passive chromium oxide layer formed on the SS surface make it impossible to use the gravimetric analysis as well as most of the electrochemical techniques to gauge its corrosion behavior in atmosphere.

The main goal of this paper is twofold: a comparison between an electrochemical test (Linear Polarization Resistance) and mass loss, and the collection of data to be compared with the ISO standard and the exposure catalogue of the EFC.

## 2. Materials and Methods

### 2.1. Steel Sample Preparation and Exposure

This paper presents the results of tests carried out on carbon steel (CS), galvanized steel (GS) and stainless steel (SS) samples. All tests have been carried out in compliance with the standard EN ISO 8565:2011—Atmospheric Corrosion Testing—General Requirements [9].

The steel coupons, which have dimensions of 100 × 150 × 2 mm according to ASTM G50 [47], are exposed on racks installed in two different locations, allowing us to study their atmospheric corrosion behavior in urban and marine environments.

**Figure 1:**
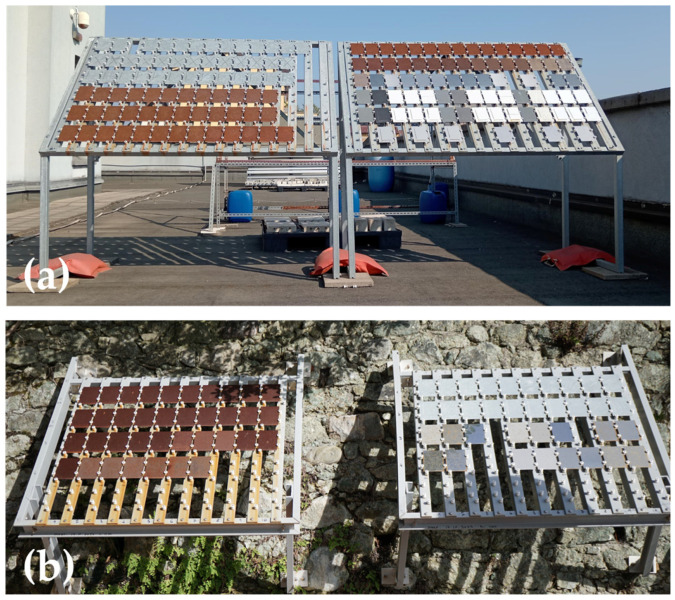
Racks exposed (**a**) on the roof of the Department of Chemistry, Materials and Chemical Engineering “Giulio Natta” of Politecnico di Milano and (**b**) outside the “MARECO” lab site (CNR—ICMATE) in Bonassola (SP).

Two racks (Figure 1a) are placed in Milan (Italy), on the roof of the Department of Chemistry, Materials and Chemical Engineering “Giulio Natta” of Politecnico di Milano (altitude + 120 m a.s.l). This exposure site is included in the “Exposure Site Catalogue” of the European Federation of Corrosion, EFC [10].

Two racks are exposed at the “MARECO” laboratory site (CNR—ICMATE) in Bonassola (SP, Liguria, Italy), directly facing the sea, about 10 m distance from the sea, at an altitude + 10 m a.s.l (Figure 1b).

All the racks, made of hot-dip galvanized steel, are oriented southerly and inclined at 45° to the vertical. Ceramic spacers have been used to avoid electrical contact between the frame and the metallic samples.

Table 2 and Table 3 summarize all the information about the exposed specimens; in the case of carbon steel, 15 samples have been sandblasted to remove any oxide traces on their surface. Stainless steel samples were exposed only in a marine atmosphere: five different grades were considered, all with a 2B finishing. The 2B surface finishing is one of the most common surface finishing for cold-rolled sheets, resulting in a brighter surface, silvery gray. It is one of the most versatile surface finishes, widely used in the chemical, paper, petrochemical and medical industries; it can also be used for external facades on buildings. The surface roughness Ra measured is in the range 0.1–0.5 μm. It is also the most common finish to ensure good corrosion resistance, smoothness and flatness.

During the whole exposure period, a large set of environmental parameters have been constantly monitored. For the Milan exposure site, relative humidity and temperature have been acquired hourly with an Ebro EBI 20 datalogger (Ingolstadt, Germany). NO_x_ and SO_x_ concentrations, precipitation and solar radiation intensities have been acquired daily from a nearby public weather station. For the Bonassola exposure site, the same data have been obtained from public weather stations instead. Chloride deposition rate has been evaluated on site periodically as indicated by ISO 9225—annex E [7].

### 2.2. Morphological and Structural Characterization

All samples underwent a periodic visual inspection each time photographs were taken. The morphology of GSl and GSx samples was characterized using a stereomicroscope Leica Wild Heerbrugg.

X-ray diffraction (XRD) was performed using a Philips PW3020 goniometer (Malvern, UK) with Cu K_α_1 radiation (1.54058 Å).

### 2.3. Electrochemical and Mass Loss Analyses

The corrosion behavior of steel specimens has been investigated through electrochemical measurements and mass loss tests, as displayed in Table 4 and Table 5.

Electrochemical tests were performed with a Metrohm Autolab PGSTAT (Herisau, Switzerland), using a local probe consisting of a flat cell made of a glass cylinder with an o-ring underneath, a saturated Ag/AgCl/KCl_sat_. reference electrode and a stainless steel, grade AISI 316, counter electrode. This measurement simulates the wet conditions experienced by the steel coupons during atmospheric exposure, such as during intense rainfall when the metal is fully wetted by rainwater. For all electrochemical measurements, a volume of 50 mL (0.05 dm^3^) was introduced into the electrochemical cell, in contact with a metal surface area of 20 cm^2^. This corresponds to 25 mm of rainfall (1 mm of rainfall = 1 dm^3^ per m^2^), representing an intense rain.

For the characterization of samples exposed in Milan, rainwater (total salinity < 10 mg/L, pH 6.6 and resistivity 600 Ω·m, chemical composition is reported in Appendix A Table A3), collected using a rain gauge placed near the rack, was used as test solution.

For specimens exposed to marine atmosphere, a 3.5% by weight NaCl solution was used, which corresponds to about 20 g/L of chlorides (the same chloride concentration of seawater in the Tyrrhenian Sea). The use of this aqueous solution is supported by the high chloride ion deposition rate on the coupons at the exposure site, due to the proximity to the sea (mean value 209 mg/(m^2^·day)—level S_2_, with maximum values of about 500 mg/(m^2^·day)). While it is well known that airborne salinity is strongly influenced by factors affecting the inland transport of sea salt—such as wind direction, wind speed, local topography and the distance from the sea—and that rainfall can leach chloride concentrations from the surface, it can be assumed that electrochemical characterization with 3.5% NaCl solution is a reliable representation of the severe exposure conditions, characterized by high humidity and a high chloride deposition rate. Considering a conservative chloride deposition rate of 1000 mg/(m^2^·day), which corresponds to the mean value of the deposition rate at level S_3_ in the ISO 9223 standard, the annual chloride ion deposition amounts to approximately 350 g/m^2^. In the solution used during electrochemical measurements, the total chloride content is 500 g/m^2^ (1 g of chloride in 0.05 dm^3^ of test solution in contact with a metal surface of 20 cm^2^).

Linear Polarization Resistance (LPR) was performed by polarizing the sample at ±20 mV with respect to the free corrosion potential (E_corr_) with a scan rate of 10 mV·min^−1^. Once the polarization resistance, *R_p_*, was extrapolated using a linear fit of the LPR results, the corrosion rate was calculated through the Stern–Geary equation:

(3) $$i_{corr}=\frac{B}{R_{p}}=\frac{b_{a}\cdot b_{c}}{2.3\cdot \left(b_{a}+b_{c}\right)}\cdot \frac{1}{R_{p}}$$

where *b_a_* and *b_c_* are the anodic and cathodic Tafel’s slopes. For CS, assuming the corrosion process of an active metal (*b_a_*~100 mV/decade) and oxygen diffusion as a cathodic process, the constant *B* is equal to 50 mV (value confirmed by experimental results). For GS, a *B* value of about 35 mV has been obtained from the Tafel plot extrapolation, performed at different exposure times measured via potentiodynamic polarization.

Potentiodynamic polarization (PDP) analyses were carried out by polarizing the sample between ±250 mV with respect to E_corr_ at a scan rate of 10 mV·min^−1^.

Mass loss tests were performed following the ASTM G1-03 (2017) [48] standard, removing corrosion products using a 50% v HCl + 3.5 g of hexamethylene tetramine solution for CS and a 100 g/L ammonium persulfate ((NH_4_)_2_S_2_O_8_) solution for GS coupons. Corrosion rate (*CR*) was then estimated as:

(4) $$CR=\frac{∆m}{S\cdot t}$$

where ∆m is the mass loss, *t* is the exposure time and *S* is the exposed surface of the sample.

Finally, measured corrosion rates have been compared to the ones forecast according to ISO 9223 and ISO 9224 [5,6].

## 3. Results

### 3.1. Environmental Parameters

**Figure 2:**
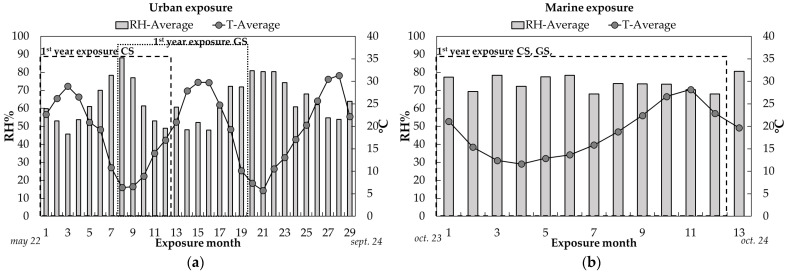
Average values of relative humidity and temperature in (**a**) Milan and (**b**) Bonassola exposure site for each month.

The average values of the relative humidity and temperature for each month of exposure are displayed in Figure 2a,b for the urban and marine environments, respectively.

The time of wetness τ (Table 6) is defined as the number of hours over the whole exposure period during which the relative humidity is larger than the critical value at which water condensation occurs. The ISO 9223 [5] recommends 80% RH as the critical threshold value; during this study, the recommended 80% threshold value was adopted for the urban exposure, where the effect of sulfur dioxide is minimal (Table 6) and no chlorides are present. For the marine environment, a second τ value was calculated, considering the time of wetness as the number of hours for which RH was higher than 60%. This assumption was made since hygroscopic salts in marine environments promote water condensation at a lower relative humidity value, thus critical RH significantly decreases [2].

The τ data for the first year of exposure are reported in Table 6 and the relative classes of time of wetness according to ISO 9223 are defined; τ at the different exposure times at which electrochemical measurements were carried out are listed in Table A4.

The average SO_x_ deposition rates are reported in Table 6 for the two exposure sites. According to these values, both environments correspond to a P_0_ level.

Concerning the Bonassola site, the chloride concentration is considered too (Table 6).

The environmental parameters of the first year of exposure for CS and GS (highlighted in Figure 2 and reported in Table 6) were used to determine the r_corr_ according to the dose–response functions suggested by ISO 9223 (Equations (5)–(8)):

- Carbon steel

(5) $$r_{corr}=1.77\cdot \;{P_{d}}^{0.52}\exp\left(0.020\cdot RH+f_{St}\right)+0.102{{\cdot S}_{d}}^{0.62}\exp\left(0.033\cdot RH+0.047\cdot T\right)$$

(6) $$f_{St}=0.150\cdot \left(T-10\right)\;when\;T\leq 10\;{}^{\circ}C;\;otherwise-0.054\cdot (T-10)$$

- Zinc

(7) $$r_{corr}=0.012\cdot \;{P_{d}}^{0.44}\exp\left(0.046\cdot RH+f_{Zn}\right)+0.0175{{\cdot S}_{d}}^{0.57}\exp\left(0.008\cdot RH+0.085\cdot T\right)$$

(8) $$f_{Zn}=0.038\cdot \left(T-10\right)\;when\;T\leq 10\;{}^{\circ}C;\;otherwise-0.071\cdot (T-10)$$

where

*r_corr_* is first-year corrosion rate of metal, expressed in micrometers per year (µm/y);

*T* is the annual average temperature, expressed in degrees Celsius (°C);

*RH* is the annual average relative humidity, expressed as a percentage (%);

*P_d_* is the annual average SO_2_ deposition, expressed in milligrams per square meter per day [mg/(m^2^·d)];

*S_d_* is the annual average Cl—deposition, expressed in milligrams per square meter per day [mg/(m^2^·d)].

### 3.2. Urban Atmosphere

#### 3.2.1. Carbon Steel

##### Free Corrosion Potential Measurements

**Figure 3:**
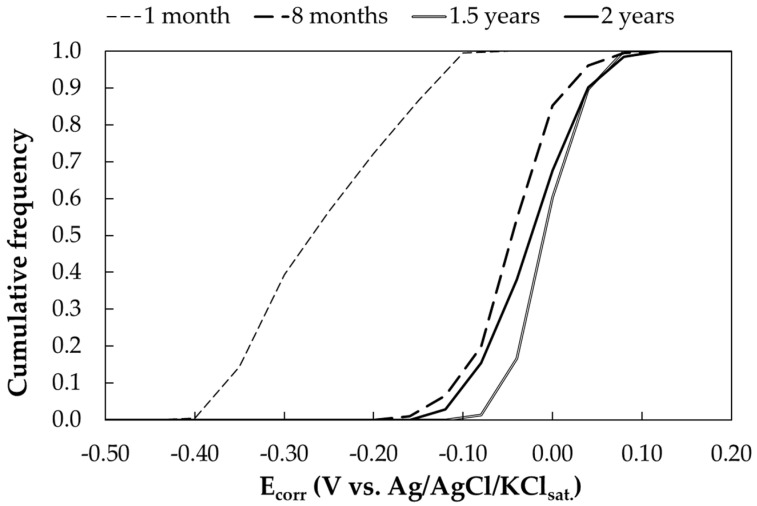
Cumulative distribution function of free corrosion potentials (E_corr_) of carbon steel samples exposed in urban environment (Milan).

Figure 3 presents the cumulative frequency curve of the free corrosion potentials as a function of time. Each curve corresponds to the distribution of a total of 90 potential measurements. After one month of exposure, carbon steel coupons show only a slight presence of corrosion products on their surface, as expected due to the short time of wetness (11%, Table A4) and exposure time. The free corrosion potential ranges from −0.4 V to −0.1 V vs. Ag/AgCl/KCl_sat_. After 8 months of exposure, a noticeable increase in free corrosion potential is observed, with values ranging from −0.1 V to 0 V vs. Ag/AgCl/KCl_sat_. This shift is attributed to the formation of corrosion products on the surface. This process leads to an increase in anodic overvoltage and a reduction in oxygen diffusion to the active metal surface. No effect of sandblasting was observed on free corrosion potential.

The effect of corrosion products in environments with medium to high resistivity and in absence of chloride ions is well documented, particularly in soil applications, where carbon steel exhibits more noble potentials due to the gradual formation of low-solubility corrosion products over time. As these corrosion products accumulate, the steel’s potential stabilizes, typically falling within the range from −0.1 V to 0 V vs. Ag/AgCl/KCl_sat_., and the standard deviation decreases. Table 7 presents the mean (50th percentile), minimum, maximum and standard deviation of the free corrosion potential in an urban environment.

**Figure A1:**
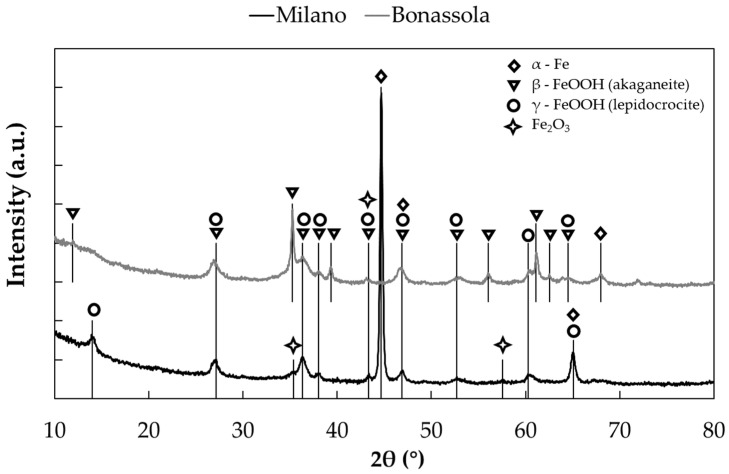
XRD diffractogram of carbon steel samples exposed in Milan and Bonassola.

The nature of these corrosion products is shown by XRD results (Figure A1), reporting that the rust layer is mainly composed of iron oxy-hydroxides, among which lepidocrocite (γ-FeOOH) is dominant. A small amount of iron oxide (Fe_2_O_3_) is observed too.

##### Corrosion Rate via Linear Polarization Resistance Measurements

The corrosion rate was calculated from the Linear Polarization Resistance values using the Stern–Geary equation, as described in Section 2.3. For each testing period, the anodic (b_a_) and cathodic (b_c_) slopes were determined from potentiodynamic polarization curves (Figure A3). For carbon steel exposed to Milan rainwater, the anodic Tafel slope ranged from about 70 to 130 mV/decade of current, increasing the exposure time. Considering oxygen reduction under diffusion–kinetic control as the main cathodic process occurring on the metal surface, the slope of the cathodic polarization curve can be considered to be much higher than the anodic slope. Consequently, the Stern–Geary constant, *B*, was taken as equal to (b_a_/2.3), with values ranging from 30 to 60 mV, at low (<1 year) and high exposure times, respectively.

Unlike immersion conditions, the corrosion rate of a metal exposed to the atmosphere is strongly influenced by the presence of moisture in contact with the metal and its ionic resistivity. In particular, electrolyte resistivity depends on the deposition of contaminants such as salts and sulfur dioxide, and represents the primary limiting factor.

In rural and urban environments, the low ionic conductivity and low corrosiveness of the moisture in contact with the metal can promote the formation of an oxide layer, which has a passivating effect and reduces the corrosion rate over time, as predicted by the dose–response equation in the ISO 9224 standard. In contrast, in industrial or marine environments, where the electrolyte has low resistivity, the corrosion rate is higher than in an urban atmosphere. Additionally, in marine environments, where chlorides deposit on the metal surface, soluble corrosion products may form, leading to weak passivation and higher corrosion rates.

In both scenarios, excluding the minor contribution from hydrogen evolution, the corrosion rate does not exceed a limiting value determined by the availability of oxygen at the metal surface. In atmospheric corrosion, the primary limiting factor is the ohmic drop in the thin electrolyte whereas, under immersion conditions, oxygen diffusion is typically the limiting factor.

Based on these considerations—and recognizing that the atmospheric corrosion rate increases with relative humidity and airborne salinity—this study calculates the corrosion rate in atmospheric conditions combining the maximum corrosion rate obtained from Linear Polarization Resistance measurements under immersion conditions with the time of wetness (*τ*), as described in Section 3.1.

The corrosion rate (in µm/year) in the atmosphere is calculated as follows:

(9) $$C.R.=1.17\cdot i_{corr}\cdot \tau \%=1.17\cdot \frac{B}{R_{p}}\cdot \tau \%$$

where 1.17 is the Faraday equivalence for iron, *i_corr_* is the corrosion rate in electrochemical units (mA/m^2^) calculated from linear polarization measurements under total immersion conditions, and *τ* is the time of wetness, equal to 11% during the first month of exposure and ranging from 28% to 33% considering longer exposure (see Table A4).

**Figure 4:**
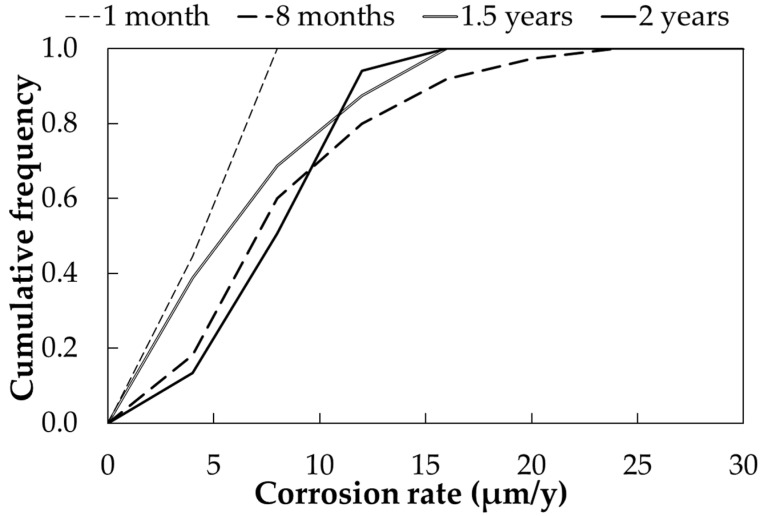
Cumulative distribution function of corrosion rates (CRs) of carbon steel samples exposed in urban environment (Milan).

Figure 4 shows the cumulative frequency curve of the corrosion rate, calculated as described, as a function of exposure time. Each curve represents the distribution of a total of 90 measurements. Excluding the distribution curve at 1 month of exposure, which shows very low corrosion rates, the corrosion rate ranges from 5 to 15 µm/year for exposure times between 8 and 24 months. The 1-month measurement should not be misunderstood: the corrosion rate under full immersion conditions is higher at early testing times because no oxide or passivation layer has formed on the metal surface. The corrosion rate after one month is between 30 and 40 mA/m^2^; however, due to the very low time of wetness (11%), the calculated corrosion rate is quite modest, below 5 µm/year as the mean value. No significant differences in mean corrosion rates were observed during the first two years of testing, as well as between sandblasted and as-received samples. On the other side, standard deviation and maximum value decrease (Table 8) from 8 to 24 months, as expected considering the relationship between corrosion attack, *D*, and time *t* (Equation (1)). Table 8 reports the mean (50th percentile), minimum, maximum and standard deviation of the corrosion rates calculated with the described approach for an urban environment.

##### Corrosion Rate by Mass Loss

The corrosion rate by mass loss was determined on three randomly selected carbon steel coupons after 10, 15, 20 and 26 months of exposure. Table 9 presents the mean values of the mass loss rate (in g/(m^2^·y)) and the corrosion penetration rate (in µm/year). The corrosion rate by mass loss decreases with exposure time, from an initial value of about 8 µm/year to approximately 2 µm/year after two years of exposure. This trend is consistent with Equation (1), where the corrosion rate is defined as

(10) $$\frac{dD}{dt}\propto t^{k}$$

with *k* = (*b* − 1).

#### 3.2.2. Galvanized Steel

##### Free Corrosion Potential Measurements

**Figure 5:**
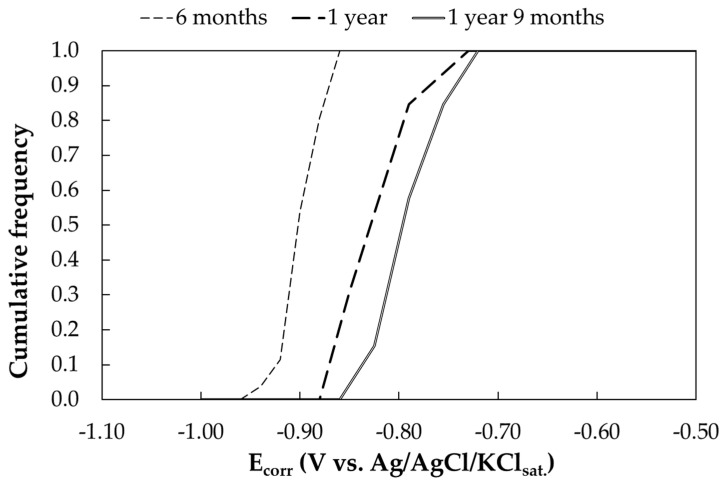
Cumulative distribution function of free corrosion potentials (E_corr_) of galvanized steel samples exposed in urban environment (Milan).

**Figure A2:**
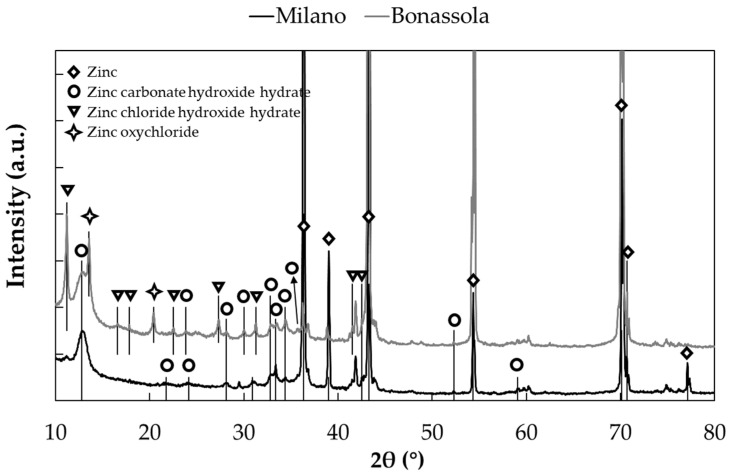
XRD diffractogram of carbon steel samples exposed in urban (Milan) and marine (Bonassola) environments.

**Figure A3:**
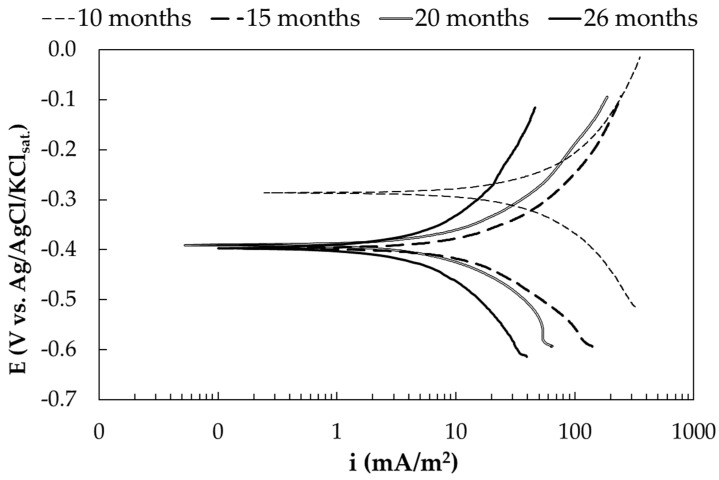
Potentiodynamic polarization curves of carbon steel samples exposed in Milan at different exposure times.

The cumulative frequency curve of the free corrosion potentials as a function of time is reported in Figure 5. Each curve corresponds to the distribution of a total of 30 potential measurements performed on the 15 exposed samples. Table 10 summarizes the mean, minimum, maximum and standard deviation of the free corrosion potential. After six months of exposure, the free corrosion potential ranges from −0.87 V to −0.94 V vs. Ag/AgCl/KCl_sat_. After 1 year, more positive potentials were measured, in the range from −0.86 V to −0.74 V vs. Ag/AgCl/KCl_sat_. This potential increase is attributed to the passivation of zinc, caused by the formation of a basic zinc carbonate layer when in contact with atmosphere due to the presence of CO_2_. The presence of zinc carbonate has been confirmed through XRD analysis (Figure A2); indeed, a layer mainly constituting zinc carbonate hydroxide hydrate (Zn_4_CO_3_(OH)_6_·H_2_O) has been observed. The third potential reading, performed at 1 year and 9 months of exposure, confirmed the trend of the potential increase.

##### Corrosion Rate by Linear Polarization Resistance Measurements

**Figure 6:**
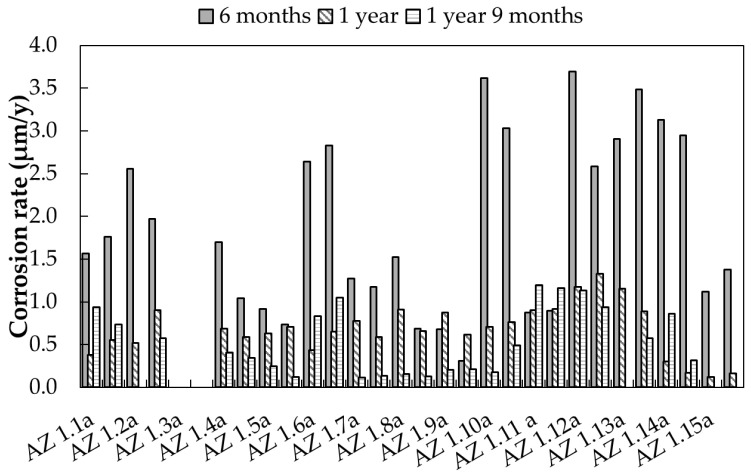
Corrosion rate values determined by means of LPR test on galvanized steel samples exposed in urban environment (Milan).

**Figure A4:**
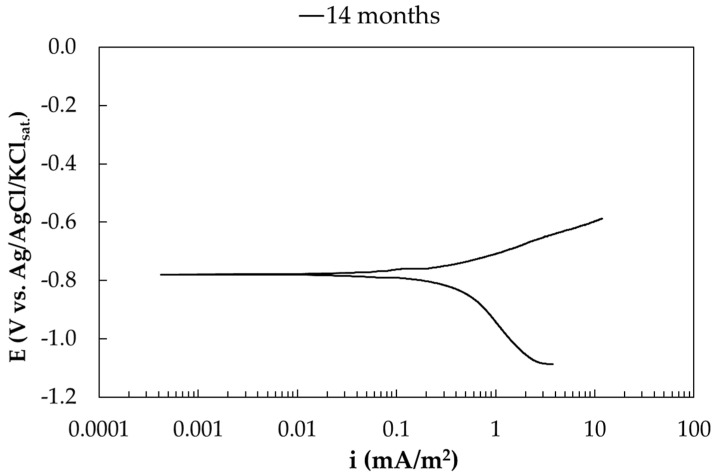
Potentiodynamic polarization curves of galvanized steel samples after 14 months of exposure in Milan.

As previously described, corrosion rate has been estimated by means of Linear Polarization Resistance measurement and by using the Stern–Geary equation, according to Equation (3). The anodic (b_a_) and cathodic (b_c_) slopes were confirmed through potentiodynamic polarization curves (Figure A4): values from about 70 to 130 mV/decade of current were measured, giving a B constant equal to 35 mV. Figure 6 reports the corrosion rate values thus determined on the 15 exposed samples: two readings have been performed on each sample. In the majority of the cases, the longer the exposure time, the lower the measured corrosion rate. The corrosion rate values measured after 21 months are, on average, three to four times lower than the values measured after 6 months. This is, as in the case of free corrosion potentials, attributed to the formation of a passive carbonate layer on the surfaces of the material.

**Figure 7:**
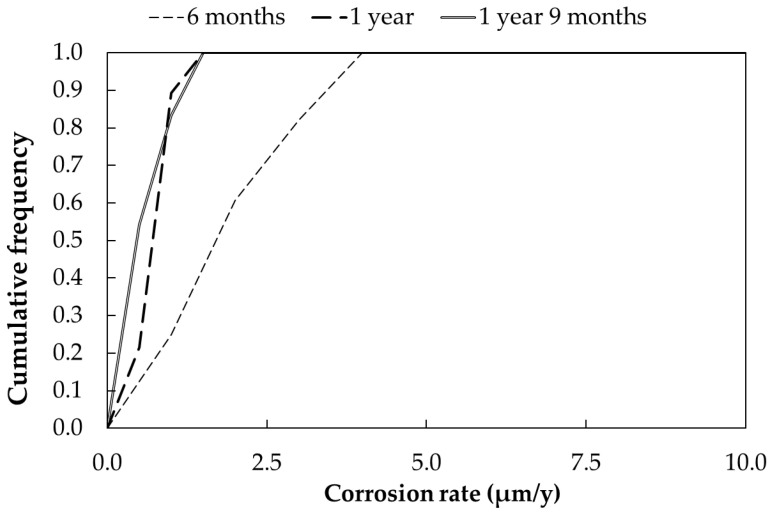
Cumulative distribution function of corrosion rates (CRs) of galvanized steel samples exposed in urban environment (Milan).

Figure 7 shows the cumulative frequency curve of the corrosion rate, also in this case calculated by considering the effect of the time of wetness, as reported in Table 11. Each curve represents the distribution of a total of 30 measurements. The corrosion rate reduction in time can be clearly observed. The mean corrosion rate is close to 2 µm/year after 6 months, while it is reduced to about 0.6–0.7 µm/year after one year of exposure.

Table 11 reports the mean (50th percentile), minimum, maximum and standard deviation of the corrosion rates calculated with the described approach for the urban environment.

##### Corrosion Rate by Mass Loss

One mass loss test was performed after 14 months of exposure. Two galvanized steel samples were tested. On a tested surface of 0.03 m^2^, a metal loss in the range of 170 mg was measured, corresponding to a mass loss rate of 0.13 mdd or to a corrosion penetration rate of 0.65 μm/year (by means of Equation (4)). The value is quite close to the mean corrosion rate measured by means of LPR after 12 and 21 months of exposure (see Table 11). A thickness loss close to 0.8 μm can be estimated after 14 months.

### 3.3. Marine Atmosphere

#### 3.3.1. Carbon Steel

Twenty carbon steel coupons, ten of which were sandblasted, were exposed to the marine atmosphere, starting in October 2023, on a rack installed in Bonassola (SP, Liguria, Italy), directly facing the sea.

##### Free Corrosion Potential Measurements

**Figure 8:**
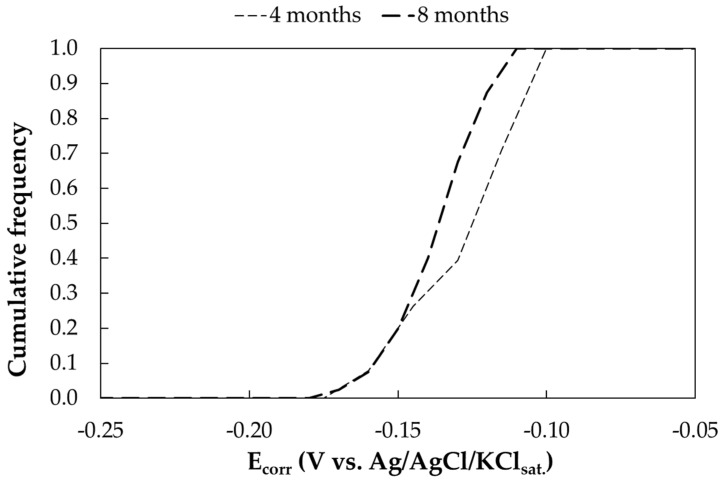
Cumulative distribution function of free corrosion potentials (E_corr_) of carbon steel samples exposed in marine environment (Bonassola).

Two free corrosion potential measurements were taken for each sample after 4 and 8 months of exposure. Figure 8 shows the cumulative frequency curve of the free corrosion potentials as a function of time. Each curve represents the distribution of a total of 40 potential measurements. The free corrosion potential ranges from −0.2 V to −0.1 V vs. Ag/AgCl/KCl_sat_. No significant variation was observed between the 4- and 8-month measurements.

When comparing carbon steel in urban and marine environments after 8 months, free corrosion potentials in the marine atmosphere are approximately 100 mV more negative than in urban environments. This can be attributed to the reduced protective effect of the corrosion products formed on the surface of the sample, due to the presence of chlorides, which prevent the metal from passivating. In the marine environment, unlike in the urban one, the effect of chlorides is twofold: (1) they increase oxide solubility, promoting a less compact corrosion product layer, and (2) they reduce the ohmic drop in the electrolyte layer in contact with the metal, leading to higher corrosion rates. No effect of sandblasting was observed on the free corrosion potential. This is confirmed via the XRD results (Figure A1), which show that the corrosion product layer formed in marine environments is dominated by akageneite (β-FeOOH), which is more soluble than the lepidocrocite which is instead dominant in urban exposure, and typically forms in chloride-rich environments.

Table 12 presents the mean (50th percentile), minimum, maximum and standard deviation of the free corrosion potential in marine environments.

##### Corrosion Rate via Linear Polarization Resistance Measurements

The corrosion rate was calculated from the Linear Polarization Resistance values using the Stern–Geary equation (Equation (3)), assuming 50 mV as the Stern–Geary constant (*B*). The general considerations regarding the use of Linear Polarization Resistance in immersion conditions to evaluate corrosion rates in urban environments can also be applied to marine environments.

Unlike urban exposure, in marine environments, the time of wetness (*τ*) was calculated according to Section 3.1.

The mean *τ* in Bonassola is 86% with a critical humidity of 60%, while it decreases to 33% when a critical humidity of 80% is used. For comparison, the ISO 9223 standard (Annex B) reports a time of wetness in the most severe conditions (humid environments, level τ_5_) greater than 63%.

**Figure 9:**
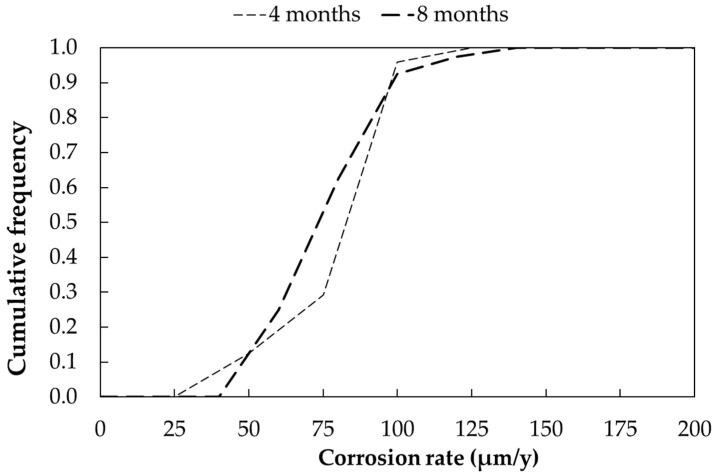
Cumulative distribution function of corrosion rates (CRs) of carbon steel samples exposed in marine environment (Bonassola).

The corrosion rate (in µm/year) in the marine atmosphere was calculated using Equation (9). Figure 9 presents the cumulative frequency curve of the corrosion rate as a function of exposure time. Each curve represents the distribution of a total of 40 measurements.

The corrosion rate ranges from about 40 to 125 µm/year (Table 13). No significant differences in mean corrosion rates were observed after 4 and 8 months, as well as between sandblasted and as-received samples.

#### 3.3.2. Galvanized Steel

##### Free Corrosion Potential Measurements

**Figure 10:**
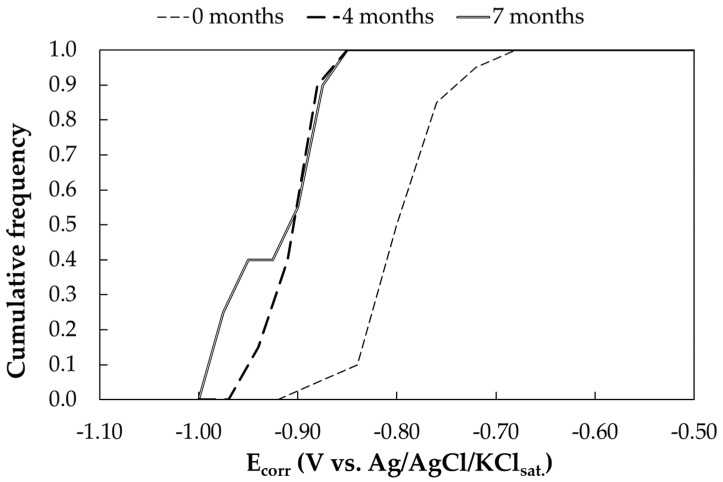
Cumulative distribution function of free corrosion potentials (E_corr_) of galvanized steel samples exposed in marine environment (Bonassola).

The cumulative frequency curve of the free corrosion potentials as a function of time is reported in Figure 10. Each curve corresponds to the distribution of a total of 20 potential measurements performed on the 10 exposed panels. Table 14 summarizes the mean, minimum, maximum and standard deviation of the free corrosion potential. Under marine exposure, the free corrosion potential after a few months of exposure decreases from values close to −800 mV Ag/AgCl/KCl_sat_. to values close to −0.900 mV vs. Ag/AgCl/KCl_sat_. Even if XRD (Figure A2) confirmed also in this case the presence of a zinc carbonate hydroxide hydrate (Zn_4_CO_3_(OH)_6_·H_2_O), zinc chloride hydroxide monohydrates (Zn_5_(OH)_8_Cl_2_·H2O) have been detected, most probably a result of the more active surface of the galvanized steel being exposed to the marine atmosphere. The results are consistent with literature on the topic [1].

##### Corrosion Rate via Linear Polarization Resistance Measurements

**Figure 11:**
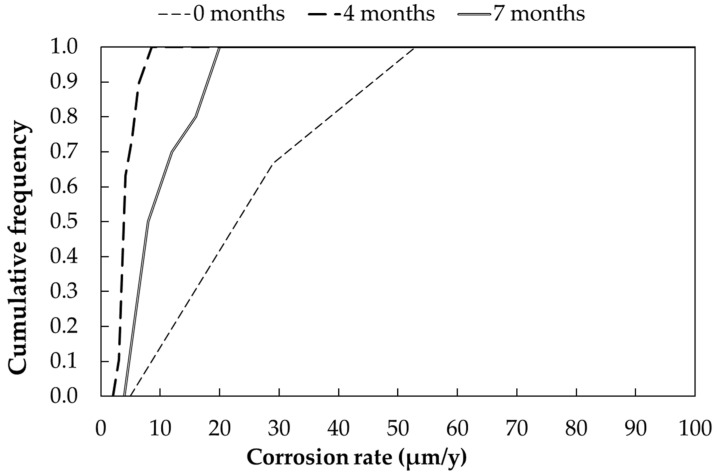
Cumulative distribution function of corrosion rates (CRs) of galvanized steel samples exposed in marine environment (Bonassola).

Figure 11 reports the corrosion rate values determined on the 10 exposed galvanized steel samples: two readings have been done on each specimen. Corrosion rate increases in time: the presence of chlorides may have weakened the passive zinc layer. Corrosion rate ranges from 2.26 to 7.75 µm/year after 4 months and from 3.81 to 18.7 µm/year after 7 months. As in the previous cases, corrosion rate determined by means of LPR has been corrected by considering the *τ* estimated as in Section 3.1. *τ* values are reported in Table 15 and are very high if compared with values determined under urban exposure.

#### 3.3.3. Stainless Steels

**Figure 12:**
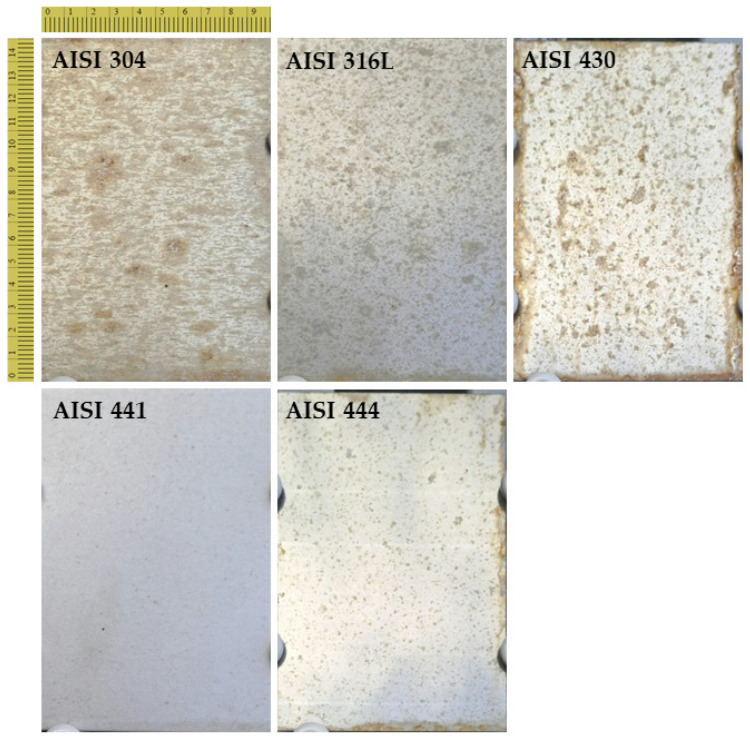
Picture of the stainless steel surface after 7 months of exposure in marine environment.

Five stainless steel grades have been exposed to marine atmosphere. After a period of 7 months of exposure (Figure 12), the following can be stated:

AISI 304—Stains appeared immediately during the first month of exposure, evenly over the entire surface. Over time, they increased in number and size. Their shape was initially elongated in the rolling direction. Only later, stains also developed in the transversal direction. At month seven, early stains, rounded in shape and with an intense red color, are observed; new stain formation, characterized by elongated shapes and a greyish color, is also observed.

AISI 316L—Stains developed mainly in the lower half of the surface, starting from the first month of exposure. Stains are characterized by a small diameter and are elongated towards the rolling direction.

AISI 430—The samples present extensive and abundant stains, mainly of an orange-reddish color. The appearance and growth of the stains occurred with very fast kinetics, being already considerably extended at the end of the first month of exposure. At each month of exposure, the samples appeared much more stained than the other alloys. At the edges of the samples, the most extensive and intense stains were detected, indicating an increased sensitivity to the prolonged retention of rainwater and atmospheric humidity polluted with chlorides. Among the considered grades, it is the most susceptible to corrosion in a marine environment.

AISI 441—The samples presented, at each month of exposure, the fewest number of stains and the least alteration of the surface, among all the samples considered. Their appearance was mainly unchanged during exposure and only a few small semi-transparent stains, of dark grey color, are visible at the seventh month.

AISI 444—A certain number of medium–small sized stains appeared in time, grey-yellowish in color and semi-transparent. No preferential orientation of the stains was noted differently from other specimens characterized by the same finish. At the edges, diffuse stains of a more intense, orange-reddish color are present. Stains appeared just after the first month of exposure, but then remained relatively stable in shape, number and size.

## 4. Discussion

### 4.1. Category of Corrosivity of the Field Sites

#### 4.1.1. Urban Field Site (Milan)

As shown in Table 8, the corrosion rates of carbon steel samples exposed in an urban environment (Milan), determined according to Equation (9), range from 4 to 8 µm/year, depending on the exposure duration. The corrosion rate (measured by mass loss) reported in Table 9 is 8.3 µm/year after 10 months of exposure and decreases to 2.4 µm/year after 15 months.

According to ISO 9223, the classification of atmospheric corrosivity can be determined either through the first-year corrosion rate measurement of standard specimens or using the dose–response function. Using the first criterion and the thresholds provided in the standard, the urban site in Milan is classified as corrosivity class C2 (first-year corrosion rate between 1.3 and 25 µm/year). Similarly, based on the dose–response function (Equation (5)) and the environmental parameters measured at the exposure site from May 2022 to May 2023 (Table 6), the first-year corrosion rate (r_corr_) is 8.65 µm/year, placing the site within the C2 category.

Similarly, the corrosion rate of galvanized steel samples exposed in an urban environment, calculated through Equation (9), is 0.68 µm/year after one year of exposure (Table 11), while the corrosion rate based on mass loss measurement is 0.65 µm/year.

According to ISO 9223, these corrosion rates correspond to an atmospheric corrosivity classification of C2 (with the first-year corrosion rate of zinc between 0.1 and 0.7 µm/year). Using the dose–response function for zinc (Equation (7)) and the environmental parameters measured at the exposure site during the first year of exposure (Table 6), the first-year corrosion rate (r_corr_) is calculated to be 0.22 µm/year, which places the site in the C2 category.

#### 4.1.2. Marine Field Site (Bonassola)

As shown in Table 13, the corrosion rates of carbon steel samples exposed to a marine atmosphere in Bonassola, determined according to Equation (9) with *τ* calculated based on a critical relative humidity of 60%, range from 79.3 to 75.8 µm/year at 4 and 8 months, respectively. Although corrosion rate data after one year of exposure are not available, a preliminary comparison with the corrosion rates specified in the ISO 9223 standard can be made.

According to ISO 9223, the marine site in Bonassola is classified as corrosivity class C4 (first-year corrosion rate between 50 and 80 µm/year), with the observed corrosion rates approaching the upper limit of this range. As a comparison, the measured corrosion rate in the first year is equivalent to a highly polluted urban–industrial aera, with SO_x_ concentrations of 80 µg/m^3^, as confirmed by Wu et al. in their 7-year-long study on carbon steel exposure to atmosphere [19].

Based on the dose–response function (Equation (5)) and the environmental parameters measured at the exposure site from October 2023 to October 2024 (Table 6), the first-year corrosion rate (r_corr_) is calculated to be 83.2 µm/year, placing the site within the C5 category (close to the lower limit of this classification).

In regard to the galvanized steel samples exposed in Bonassola, the corrosion rate is 4.4 µm/year after four months and 9.4 µm/year after eight months of exposure (Table 15). Actually, the unexpected increase in corrosion rate over time warrants further investigation. These corrosion rates correspond to an atmospheric corrosivity classification of C5 (first-year corrosion rate of zinc between 4.2 and 8.4 µm/year). Using the dose–response function for zinc (Equation (7)), the first-year corrosion rate (r_corr_) is 3.6 µm/year, which places the site within the C4 category. In similar environmental conditions, the corrosion rate of zinc was found to be 8.4 µm/year in Brest and 3.7 µm/year in Cadiz [49].

Table 16 summarizes the experimental corrosion rate measurements and reports the related corrosivity categories according to the ISO 9223 standard.

**Figure 13:**
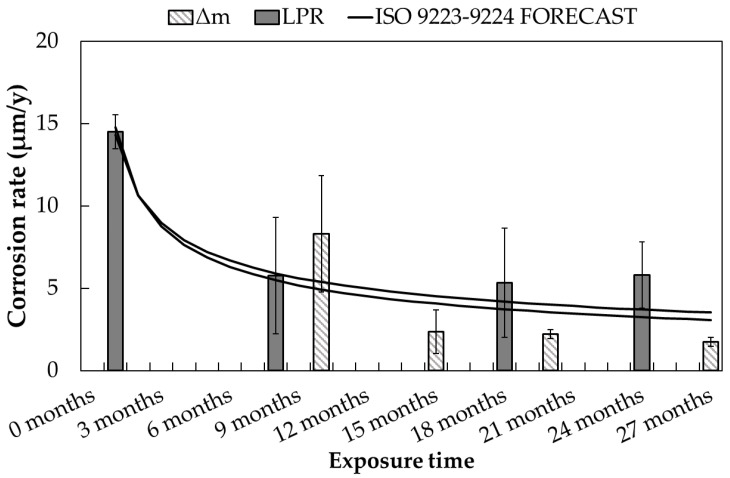
Time evolution of carbon steel corrosion rate in urban environment: comparison between the ISO standard prediction and the experimental data.

Once the corrosion rate in the first year of the exposure is obtained, it is possible to derive the corrosion rate evolution over time by exploiting the derivative in time of Equation (1). Thus, the corrosion rate values measured by means of mass loss and Linear Polarization Resistance can be compared with the ISO standard prediction. A good agreement is observed between the observed data and the standard forecast for both CS and GS in both environments. The detailed comparison is given in Figure 13 for the carbon steel specimens exposed in the urban atmosphere.

### 4.2. Corrosion Rate in Urban and Marine Atmosphere: Benchmark Analysis with European Field Sites

#### 4.2.1. Urban Environment

**Figure 14:**
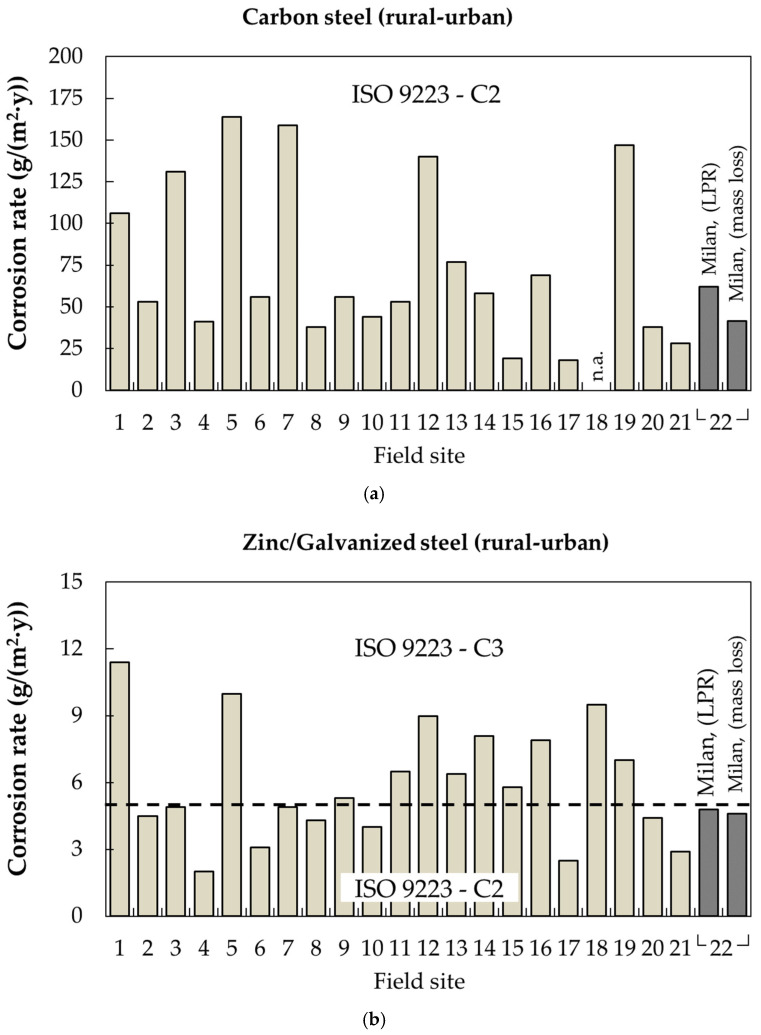
Benchmark analysis of the corrosion rates of carbon steel and zinc at urban field sites from the Exposure Site Catalogue of the European Federation of Corrosion (EFC). (**a**) Corrosion rate of carbon steel in urban environment; (**b**) Corrosion rate of galvanized steel in urban environment (**1**: AT, Linz; **2**: CZ, Kasperske Hory; **3**: CZ, Kopisty; **4**: CZ, Kralupy; **5**: CZ, Ostrava; **6**: CZ, Prague; **7**: DE, Berlin A103; **8**: DE, Berlin B1; **9**: DE, Berlin BAM; **10**: DE, Horstwalde; **11**: ES, Barcelona; **12**: FR, Le Croisty; **13**: GR, Athens; **14**: NO, Birkenes; **15**: NO, Oslo; **16**: NO, Svanvik; **17**: PL, Katowice; **18**: PT, Lisbon; **19**: PT, Lumiar; **20**: SE, Gällivare; **21**: SE, Ryda; **22**: IT, Milano; the GPS coordinates are available in the EFC catalogue).

Experimental corrosion rates of carbon steel and galvanized steel in Milan are compared in Figure 14 with data from the Exposure Site Catalogue of atmospheric corrosion, provided by the European Federation of Corrosion (EFC). The catalogue includes 22 sites in rural/urban environments and 16 sites in marine environments (urban or industrial). These field sites differ in terms of climatic parameters such as temperature, relative humidity, precipitation and the presence of gaseous pollutants. For this comparison, in both environments and for carbon steel and galvanized steel, the corrosion rate values are expressed in g/(m^2^·y) for comparison purposes with the Exposure Site Catalogue.

For carbon steel exposed in Milan (site 22 in Figure 14a), two corrosion rates are reported: one calculated from Linear Polarization Resistance (LPR) and the other derived from mass loss testing (in the latter case, the mean value calculated at 10 and 15 months is shown). The comparison with corrosion rates measured at other field sites shows good agreement and all of them can be classified with C2 corrosivity (corrosion rate between 10 and 200 g/(m^2^·y)). The mean corrosion rate across all 22 urban European field sites is 73 g/(m^2^·y), which aligns well with the corrosion rate measured in Milan and with the corrosion rate predicted by the dose–response function—68 g/(m^2^·y)—using Milan’s environmental parameters.

Concerning some specific value found in different exposures, from the data in the EFC catalogue, it is evident that site 5 (Ostrava) is urban/industrial, site 7 (Berlin A103) is near to the highway and subjected to significant chloride deposition, site 12 is characterized by a very high time of wetness, and sites 18 and 19, located in Portugal, are defined as urban but are characterized by a significant concentration of SO_X_.

Figure 14b shows the corrosion rates in the same field sites, but for zinc (hot-dip galvanized steel in our experimental campaign). Of the 22 field sites, 11 are classified as C3 corrosivity (corrosion rates between 5 and 15 g/(m^2^·y) according to ISO 9223). The remaining sites fall into the C2 corrosion category (corrosion rates between 0.7 and 5 g/(m^2^·y)). The mean corrosion rate across all the field sites is 5.8 g/(m^2^·y)—falling under the C3 category—and the corrosion rate predicted by the dose–response function, using Milan’s environmental parameters, is 1.56 g/(m^2^·y).

The corrosion rate predicted by the dose–response function appears to underestimate the actual corrosion rate for zinc in Milan.

#### 4.2.2. Marine Environment

**Figure 15:**
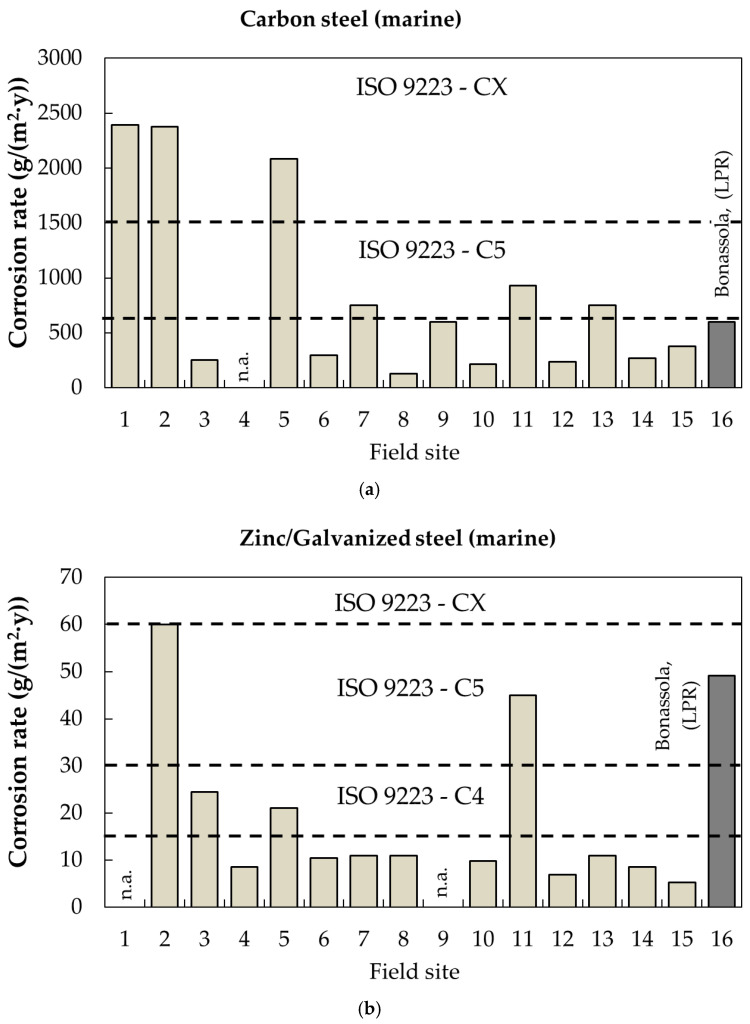
Benchmark analysis of the corrosion rates of carbon steel and zinc at marine field sites from the Exposure Site Catalogue of the European Federation of Corrosion (EFC). (**a**) Corrosion rate of carbon steel in marine environment; (**b**) Corrosion rate of galvanized steel in marine environment (**1**: DE, Helgoland IFAM; **2**: DE, Helgoland Seawater; **3**: DE, Helgoland Südhafen; **4**: DE, Helgoland Uplands; **5**: DE, Helgoland Seawater IFAM; **6**: DE, Helgoland-Westkaje; **7**: FR, Brest; **8**: IT, Genoa; **9**: NO, Tananger; **10**: PT, Alfanzina; **11**: PT, Sines; **12**: SE, Bohus-Malmön Kattesand; **13**: SE, Bohus-Malmön Kvarnvik; **14**: SE, Bohus-Malmön Kvarnvik 3; **15**: SE, Kristineberg; **16**: IT, Bonassola; the GPS coordinates are available in the EFC catalogue).

Corrosion rates of carbon steel and galvanized steel in a marine environment (Bonassola) are compared in Figure 15 with data from the Exposure Site Catalogue of the EFC. A total of 16 sites are considered.

For carbon steel exposed in Bonassola (site 16 in Figure 15a), the corrosion rate calculated from Linear Polarization Resistance (LPR) at 8 months is shown. The corrosion rate across different marine sites varies widely, ranging from 200 to 2300 g/(m^2^·y), depending on climatic parameters and factors affecting chloride deposition, such as proximity to the coast, wind intensity and direction and other environmental conditions. The mean corrosion rate is 817 g/(m^2^·y) (C5 category). In Bonassola, the value is around 600, so in good agreement. Concerning some specific value found in different exposures, from the data in the EFC catalogue it is evident that site number 11 can be defined as marine–industrial due to the high concentration of SO_x_.

Figure 15b shows the corrosion rates for zinc/hot-dip galvanized steel at the same field sites. For the Bonassola site, the mean corrosion rate, measured using the LPR technique after 4 and 8 months, is reported as 49.2 g/(m^2^·y).

Of the 13 field sites where both carbon steel and zinc corrosion rates are available, the corrosivity category is the same for both metals at two sites (#2 and #3). At seven sites (#6, 8, 10, 11, 12, 14 and 15), the category based on zinc data is one level lower (e.g., from C4 to C3) than the category based on carbon steel corrosion rates. At three sites (#5, 7 and 13), the zinc-based corrosivity category is two levels lower (e.g., from CX to C4) compared to the category based on carbon steel corrosion rates. In Bonassola, the corrosivity category for zinc is C5 while, for carbon steel, it is C4.

The mean corrosion rate, considering all field sites, is 20 g/(m^2^·y) (C4 category). Unlike in urban environments, a general trend in marine environments is that the corrosivity category for zinc is lower than that for carbon steel, based on the corrosion rates observed for each metal.

In the experiments carried out during the present work, the ratio between the average corrosion rate in marine versus urban exposure for carbon steel is higher than 10, while for zinc it is about 3. This could explain the differences in the corrosivity categories found between the two metals, especially for marine exposure. In the case of zinc, the reduced difference between the two exposures can be explained on the basis of the corrosion product: according to the literature, also in a marine environment, this is mainly composed of zinc basic carbonate (similar to in urban exposure), with minor amounts of zinc basic chlorides [49].

## 5. Conclusions

The present work addresses the atmospheric corrosion of carbon steel, galvanized steel and stainless steel exposed in both urban (Milan, Lombardia) and marine (Bonassola, Liguria) environments. The corrosion rate has been estimated both through Linear Polarization Resistance (LPR) and mass loss tests, and results have been compared with values provided by the ISO standard and by other exposure sites belonging to the EFC Exposure Sites Catalogue.

The collection of environmental data allowed the application of the ISO 9223 dose–response functions, thus placing the urban site in the C2 corrosivity category for both CS and GS, and the marine site in the C4 class for GS and in the C5 one for CS.

The tests carried out on carbon steel (CS) led to the estimation of a corrosion rate in good agreement with the value obtained using the dose–response function. For the coupons exposed to the urban environment, the different measurement methods yielded values belonging to corrosivity class C2 while, for the marine environment, a C4 class was found.

In the case of hot-dip galvanized steel (GS), the data calculated through LPR and mass loss experiments match those resulting from the dose–response method only for the urban environment, for which a C2 corrosivity class is determined. Concerning the corrosion rate calculated for the marine exposure site, the measured values appear slightly larger than those derived from ISO 9223, and fall into the C5 category instead of the C4 one. XRD measurements suggest that this trend may be due to the nature of the corrosion products formed on GS, mainly containing a certain amount of zinc basic chlorides that do not offer protection against corrosion, different from the zinc carbonate produced in an urban atmosphere.

Finally, benchmark analysis shows that the measured corrosion rates are aligned with results obtained from analogous sites of the EFC catalogue.

## Figures and Tables

**Table 1:** First-year corrosion rates (r_corr_) of carbon steel and zinc for different corrosivity categories [5].

Corrosivity Category	Corrosivity	Unit	Carbon Steel	Zinc	Typical Environments
C1	Very Low	g/(m^2^y) μm/y	r_corr_ ≤ 10 r_corr_ ≤ 1.3	r_corr_ ≤ 0.7 r_corr_ ≤ 0.1	Dry or cold zone, atmospheric environment with very low pollution and time of wetness
C2	Low	g/(m^2^y) μm/y	10 < r_corr_ ≤ 200 1.3 < r_corr_ ≤ 25	0.7 < r_corr_ ≤ 5 0.1 < r_corr_ ≤ 0.7	Temperate zone, atmospheric environment with low pollution (SO_2_ < 5 µg/m^3^), e.g., rural areas, small towns; dry or cold zone, atmospheric environment with short time of wetness
C3	Medium	g/(m^2^y) μm/y	200 < r_corr_ ≤ 400 25 < r_corr_ ≤ 50	5 < r_corr_ ≤ 15 0.7 < r_corr_ ≤ 2.1	Temperate zone, atmospheric environment with medium pollution (SO_2_: 5 µg/m^3^ to 30 µg/m^3^) or some effect of chlorides
C4	High	g/(m^2^y) μm/y	400 < r_corr_ ≤ 650 50 < r_corr_ ≤ 80	15 < r_corr_ ≤ 30 2.1 < r_corr_ ≤ 4.2	Temperate zone, atmospheric environment with high pollution (SO_2_: 30 µg/m^3^ to 90 µg/m^3^) or substantial effect of chlorides
C5	Very high	g/(m^2^y) μm/y	650 < r_corr_ ≤ 1500 80 < r_corr_ ≤ 200	30 < r_corr_ ≤ 60 4.2 < r_corr_ ≤ 8.4	Temperate and subtropical zone, atmospheric environment with very high pollution (SO_2_: 90 µg/m^3^ to 250 µg/m^3^) and/or significant effect of chlorides
CX	Extreme	g/(m^2^y) μm/y	1500 < r_corr_ ≤ 5500 200 < r_corr_ ≤ 700	60 < r_corr_ ≤ 180 8.4 < r_corr_ ≤ 25	Subtropical and tropical zone (very high time of wetness), atmospheric environment with very high SO_2_ pollution (higher thana 250 μg/m^3^) including accompanying and production factors and/or strong effect of chlorides

**Table 2:** Steel samples exposed in Milan (urban environment).

Steel Type	Label	Number	Surface Finishing
Carbon steel	CSa	30	As received
	CS	15	Sandblasted
Galvanized steel	GS	15	As received

**Table 3:** Steel samples exposed in Bonassola (marine environment).

Steel Type	Label	Number	Surface Finishing
Carbon steel	CSa	10	As received
	CS	10	Sandblasted
Galvanized steel	GS	10	As received
Stainless steel	304 *	1	2B **
	316L *	1	2B **
	430 *	2	2B **
	441 *	2	2B **
	444 *	2	2B **

* The nominal chemical composition of SSs is reported in Appendix A Table A1. ** Description of surface finishing is reported in Appendix A Table A2.

**Table 4:** Tests performed on steel samples exposed in Milan.

Type of Test	Sample	Time of Exposure (Months)
XRD	CSa + CS	0, 10, 18
	GS	0, 12
LPR	CSa + CS	1, 8, 18, 24
	GS	6, 12, 20
PDP	CSa + CS	10, 15, 20, 26
	GS	14
Mass loss	CSa + CS	10, 15, 20, 26
	GS	14

**Table 5:** Corrosion tests performed on steel samples exposed in Bonassola (SP).

Type of Test	Sample	Time of Exposure (Months)
XRD	CSa + CS	0, 4
	GS	0, 4
LPR	CSa + CS	4, 8
	GS	0, 4, 7
	SS	0, 7

**Table 6:** Average values of environmental parameters measured in the first year of exposure and determination of the pollution categories according to EN ISO 9223:2012.

Parameter	CS—Urban	GS—Urban	Marine
Period	22–23 May	22–23 December	23–24 October
T (°C)	17.2	18.1	18.4
RH%	63	61	74
SO_2_ (µg/m^3^)	2.3 (P_0_)	2.2 (P_0_)	3.2 (P_0_)
Cl^−^ (mg/m^2^·d)	-	-	209 (S_2_)
NO_x_ (µg/m^3^)	29.2	28.0	13.9
Rainfall (mm/y)	574	853	1317
Total radiation (W/m^2^)	165	163	161
Time of wetness (60%)	-	-	0.86 (τ_5_)
Time of wetness (80%)	0.27 (τ_3_)	0.23 (τ_3_)	0.33 (τ_4_)

**Table 7:** Free corrosion potentials (E_corr_) of carbon steel samples exposed in urban environment (Milan).

Exposure Time (Months)	E_corr_ (V vs. Ag/AgCl/KCl_sat_)			
	Mean	Standard Dev.	Min.	Max.
1	−0.260	0.082	−0.414	−0.098
8	−0.045	0.047	−0.161	0.083
18	−0.005	0.034	−0.090	0.068
24	−0.023	0.052	−0.138	0.090

**Table 8:** Corrosion rate (CR) of carbon steel samples exposed in urban environment (Milan), calculated according to Equation (9).

Exposure Time (Months)		CR (µm/year)			
	*τ* %	Mean	Standard Dev.	Min.	Max.
1	11	4.1	1.0	1.4	7
8	33	7.9	4.9	0.1	23.3
18	28	6.2	3.9	0.1	15.3
24	29	7.4	2.9	1.1	12.4

**Table 9:** Mass loss rate and penetration rate of corrosion of carbon steel samples exposed in urban environment (Milan).

Exposure Time (Months)	Mass Loss Rate (g/(m^2^·y))	Corrosion Penetration Rate (µm/y)
10	64.7	8.3
15	18.4	2.4
20	17.3	2.2
26	13.6	1.7

**Table 10:** Free corrosion potentials (E_corr_) of galvanized steel samples exposed in urban environment (Milan).

Exposure Time (Months)	E_corr_ (V vs. Ag/AgCl/KCl_sat_)			
	Mean	Standard Dev.	Min.	Max.
6	−0.900	0.018	−0.940	−0.870
12	−0.824	0.034	−0.865	−0.740
21	−0.793	0.030	−0.850	0.740

**Table 11:** Corrosion rate (CR) of galvanized steel samples exposed in urban environment (Milan), calculated according to Equation (9).

Exposure Time (Months)		CR (µm/Year)			
	*τ* %	Mean	Standard Dev.	Min.	Max.
6	25	1.89	1.01	0.31	3.69
12	31	0.68	0.33	0.12	1.33
21	28	0.54	0.38	0.11	1.19

**Table 12:** Free corrosion potentials (E_corr_) of carbon steel samples exposed in marine environment (Bonassola).

Exposure Time (Months)	E_corr_ (V vs. Ag/AgCl/KCl_sat_)			
	Mean	Standard Dev.	Min.	Max.
4	−0.128	0.020	−0.170	−0.100
8	−0.137	0.015	−0.173	−0.114

**Table 13:** Corrosion rate (CR) of carbon steel samples exposed in marine environment (Bonassola), evaluated according to Equation (9).

Exposure Time (Months)		CR (µm/Year)			
	*τ* %	Mean	Standard Dev.	Min.	Max.
4	88	79.3	17.3	45.7	106.3
8	85	75.8	19.8	40.4	122.5

**Table 14:** Free corrosion potentials (E_corr_) of galvanized steel samples exposed in marine environment (Bonassola).

Exposure Time (Months)	E_corr_ (V vs. Ag/AgCl/KCl_sat_)			
	Mean	Standard Dev.	Min.	Max.
0	−0.796	0.044	−0.880	−0.681
4	−0.908	0.022	−0.943	−0.874
7	−0.925	0.049	−0.999	0.866

**Table 15:** Corrosion rate (CR) of galvanized steel samples exposed in marine environment (Bonassola), calculated according to Equation (9).

Exposure Time (Months)		CR (µm/Year)			
	*τ* %	Mean	Standard Dev.	Min.	Max.
0	n.d.	26	10.7	9.56	48.84
4	88	4.42	1.48	2.26	7.75
7	85	9.37	5.31	3.81	18.70

**Table 16:** Experimental corrosion rate measurements and corrosivity categories according to ISO 9223.

Field Site	Environmental			Material	Corrosion Rate [µm/Year]			Category of Corrosivity
	T (°C)	RH %	*τ* %		By LPR	By Mass Loss	Dose–Response (ISO 9223)	Standard Coupons
Urban	17.2	63	27	CS	4–8	8.3 (10 months)	8.65	C2
				GS	0.68	0.65 (12 months)	0.22	C2
Marine	18.4	74	86	CS	79.3–75.8	---	83.2	C4
				GS	4.4–9.4	---	3.6	C5

## Data Availability

The raw data supporting the conclusions of this article will be made available by the authors on request since the experiments are forecast for 20 years and the study is currently ongoing.

## Appendix A

**Table A1:** Microstructure (A = austenitic, F = ferritic, D = duplex), chemical composition and PREN (Pitting Resistance Equivalent Number) of SS samples.

Steel Type	Microstructure	Chemical Composition						PREN
		C	Cr	Ni	Mo	Mn	N	
304	A	0.07	18.46	7.78	-	0.74	≤0.1	20.06
316L	A	0–0.03	18.07	12	2	1.74	≤0.1	26.27
430	F	0.08	16.06	0.39	-	-	-	16.06
441	F	0–0.03	15.78	-	-	0.42	-	15.78
444	F	0–0.025	17.03	-	2.07	0.14	≤0.035	24.42

**Table A2:** Surface finishing of SS samples.

Surface Finishing	
2B	Cold rolled, heat treated, pickled, subjected to subsequent light rolling

**Table A3:** Chemical composition of rainwater collected in Milan and used for electrochemical testing.

Chemical Composition [ppm]					
Ca^2+^	K^+^	Na^+^	Cl^−^	NO_3_^−^	SO_4_^2−^
2.29	0.28	1.39	2.30	5.94	2.74

**Table A4:** Time of wetness measured at the different measurement times.

Period	CS—Urban	GS—Urban	Marine (60%)	Marine (80%)
1 month	0.11	-	-	-
4 months	-	-	0.88	0.55
6 months	-	0.25	-	-
8 months	0.33	-	0.85	0.51
12 months	-	0.31	-	-
18 months	0.28	-	-	-
21 months	-	0.28	-	-
24 months	0.29	-	-	-

## References

[1] Leygraf C. (2016). Atmospheric Corrosion.

[2] Pedeferri P. (2018). Corrosion Science and Engineering.

[3] Knotkova D., Kreislova K., Dean S. (2012). International Atmospheric Exposure Program: Summary of Results.

[4] Morcillo M. (1995). Atmospheric Corrosion in Ibero-America: The MICAT Project.

[5] (2012). Corrosion of Metals and Alloys—Corrosivity of Atmospheres—Classification, Determination and Estimation.

[6] (2012). Corrosion of Metals and Alloys—Values for the Corrosivity Categories.

[7] (2012). Corrosion of Metals and Alloys-Corrosivity of Atmospheres-Measurement of Environmental Parameters Affecting Corrosivity of Atmospheres.

[8] (2012). Corrosion of Metals and Alloys-Corrosivity of Atmospheres-Determination of Corrosion Rate of Standard Specimens for the Evaluation of Corrosivity.

[9] (2011). Metals and Alloys—Atmospheric Corrosion Testing—General Requirements.

[10] https://corrosion-sites.com/.

[11] Albrecht P., Hall T.T. (2003). Atmospheric Corrosion Resistance of Structural Steels. Perspectives in Civil Engineering: Commemorating the 150th Anniversary of the American Society of Civil Engineers.

[12] de la Fuente D., Díaz I., Simancas J., Chico B., Morcillo M. (2011). Long-term atmospheric corrosion of mild steel. Corros. Sci..

[13] Castaño J.G., Botero C.A., Restrepo A.H., Agudelo E.A., Correa E., Echeverría F. (2010). Atmospheric corrosion of carbon steel in Colombia. Corros. Sci..

[14] Misawa T., Asami K., Hashimoto K., Shimodaira S. (1974). The mechanism of atmospheric rusting and the protective amorphous rust on low alloy steel. Corros. Sci..

[15] Asami K., Kikuchi M. (2003). In-depth distribution of rusts on a plain carbon steel and weathering steels exposed to coastal-industrial atmosphere for 17 years. Corros. Sci..

[16] Antunes R.A., Costa I., de Faria D.L.A. (2003). Characterization of corrosion products formed on steels in the first months of atmospheric exposure. Mater. Res..

[17] Kamimura T., Nasu S., Tazaki T., Kuzushita K., Morimoto S. (2002). Mössbauer spectroscopic study of rust formed on a weathering steel and a mild steel exposed for a long term in an industrial environment. Mater. Trans..

[18] Oh S.J., Cook D.C., Townsend H.E. (1999). Atmospheric corrosion of different steels in marine, rural and industrial environments. Corros. Sci..

[19] Wu H., Luo Y., Zhou G. (2023). The Evolution of the Corrosion Mechanism of Structural Steel Exposed to the Urban Industrial Atmosphere for Seven Years. Appl. Sci..

[20] Cook D.C. (2005). Spectroscopic identification of protective and non-protective corrosion coatings on steel structures in marine environments. Corros. Sci..

[21] Thierry D., Persson D., Le Bozec N. (2018). Atmospheric corrosion of zinc and zinc alloyed coated steel. Encyclopedia of Interfacial Chemistry: Surface Science and Electrochemistry.

[22] Elsner C.I., Seré P.R., Di Sarli A.R. (2012). Atmospheric corrosion of painted galvanized and 55% Al-Zn steel sheets: Results of 12 years of exposure. Int. J. Corros..

[23] de la Fuente D., Castaño J.G., Morcillo M. (2007). Long-term atmospheric corrosion of zinc. Corros. Sci..

[24] (2023). Stainless Steels—Part 1: List of Stainless Steels.

[25] Frankelt G.S. (1998). Pitting Corrosion of Metals: A Review of the Critical Factors. J. Electrochem. Soc..

[26] Uhlig H., Revie R.W. (2011). Uhlig’s Corrosion Handbook.

[27] Stefec R., Franz F. (1978). A study of the pitting corrosion of cold-worked stainless steel. Corros. Sci..

[28] Fontana M.G. (1987). Corrosion Engineering; McGraw-Hill Book Company.

[29] Szklarska-Smialowska Z. (1986). Pitting Corrosion of Metals.

[30] Sedriks J. (1979). Corrosion of Stainless Steels.

[31] Grubb J.F., Ludlum A., Fritz J.D., Stainless T.M.R. (2018). Corrosion of Wrought Stainless Steels. ASM Handb..

[32] Sedriks A.J. (1986). Effects of alloy composition and microstructure on the passivity of stainless steels. Corrosion.

[33] Iversen A., Leffler B. (2010). Aqueous corrosion of stainless steels. Shreir’s Corrosion.

[34] Ilevbare G.O., Burstein G.T. (2001). Role of alloyed molybdenum in the inhibition of pitting corrosion in stainless steels. Corros. Sci..

[35] Peguet L., Malki B., Baroux B. (2007). Influence of cold working on the pitting corrosion resistance of stainless steels. Corros. Sci..

[36] Rhouma A.B., Braham C., Fitzpatrick M.E., Lédion J., Sidhom H. (2001). Effects of surface preparation on pitting resistance, residual stress, and stress corrosion cracking in austenitic stainless steels. J. Mater. Eng. Perform..

[37] Messinese E., Casanova L., Paterlini L., Capelli F., Bolzoni F., Ormellese M., Brenna A. (2022). A comprehensive in-vestigation on the effects of surface finishing on the resistance to localized corrosion of stainless steel. Metals.

[38] Bellezze T., Viceré A., Giuliani G., Sorrentino E., Roventi G. (2018). Study of localized corrosion of AISI 430 and AISI 304 batches having different roughness. Metals.

[39] Rodríguez-Yáñez J.E., Batlle S.F., Sanabria-Chinchilla J., Rojas-Marín J.F. (2023). Combined effect of the exposure angle and face orientation on the atmospheric corrosion behavior of low carbon steel. Electrochim. Acta.

[40] Xia D.H., Deng C.M., Macdonald D., Jamali S., Mills D., Luo J.L., Strebl M.G., Amiri M., Jin W., Song S. (2022). Electrochemical measurements used for assessment of corrosion and protection of metallic materials in the field: A critical review. J. Mater. Sci. Technol..

[41] Macdonald D.D. (1978). An Impedance Interpretation of Small Amplitude Cyclic Voltammetry: I. Theoretical Analysis for a Resistive-Capacitive System. J. Electrochem. Soc..

[42] Wang S., Zhang J., Gharbi O., Vivier V., Gao M., Orazem M.E. (2021). Electrochemical impedance spectroscopy. Nat. Rev. Methods Primers.

[43] Bosch R.W., Bogaerts W.F. (1996). Instantaneous Corrosion Rate Measurement with Small-Amplitude Potential Intermodulation Techniques. Corrosion.

[44] Bosch R.W. (2001). Electrochemical frequency modulation: A new electrochemical technique for online corrosion monitoring. Corrosion.

[45] Abdel-Rehim S.S., Khaled K.F., Abd-Elshafi N.S. (2006). Electrochemical frequency modulation as a new technique for monitoring corrosion inhibition of iron in acid media by new thiourea derivative. Electrochim. Acta.

[46] Mameno S.H., Pettersson R., Leygraf C., Wegrelius L. (2016). Atmospheric corrosion resistance of stainless steel: Results of a field exposure program in the middle-east. Berg. Huttenmännische Monatshefte.

[47] (2020). Standard Practice for Conducting Atmospheric Corrosion Tests on Metals.

[48] (2017). Practice for Preparing, Cleaning, and Evaluating Corrosion Test Specimens.

[49] Persson D., Thierry D., Karlsson O. (2017). Corrosion and corrosion products of hot dipped galvanized steel during long term atmospheric exposure at different sites world-wide. Corros. Sci..

